# Immature Dendritic Cell Therapy Confers Durable Immune Modulation in an Antigen-Dependent and Antigen-Independent Manner in Nonobese Diabetic Mice

**DOI:** 10.1155/2018/5463879

**Published:** 2018-02-14

**Authors:** Jeannette Lo, Chang-Qing Xia, Ruihua Peng, Michael J. Clare-Salzler

**Affiliations:** Department of Pathology, Immunology and Laboratory Medicine, Center for Immunology and Transplantation, University of Florida, Gainesville, FL 32610, USA

## Abstract

Dendritic cell (DC) immunotherapy has been effective for prevention of type 1 diabetes (T1D) in NOD mice but fails to protect if initiated after active autoimmunity. As autoreactivity expands inter- and intramolecularly during disease progression, we investigated whether DCs unpulsed or pulsed with *β* cell antigenic dominant determinants (DD), subdominant determinants (SD), and ignored determinants (ID) could prevent T1D in mice with advanced insulitis. We found that diabetes was significantly delayed by DC therapy. Of interest, DCs pulsed with SD or ID appeared to provide better protection. T lymphocytes from DC-treated mice acquired spontaneous proliferating capability during *in vitro* culture, which could be largely eliminated by IL-2 neutralizing antibodies. This trend maintained even 29 weeks after discontinuing DC therapy and appeared antigen-independent. Furthermore, CD4+Foxp3+ T regulatory cells (Tregs) from DC-treated mice proliferated more actively *in vitro* compared to the controls, and Tregs from DC-treated mice showed significantly enhanced immunosuppressive activities in contrast to those from the controls. Our study demonstrates that DC therapy leads to long-lasting immunomodulatory effects in an antigen-dependent and antigen-independent manner and provides evidence for peptide-based intervention during a clinically relevant window to guide DC-based immunotherapy for autoimmune diabetes.

## 1. Introduction

Type 1 diabetes (T1D) is an autoimmune disorder resulting from the loss of self-tolerance to pancreatic islet *β* cell autoantigens. Efforts to redirect the immune response toward tolerance through peptide or whole autoantigen-based therapy have been shown to be effective in autoimmune mouse models, but have met with considerable setbacks in human studies [1–8]. Difficulties in translating the appropriate tolerizing antigen dose combined with the risk of activating or enhancing autoimmunity have delayed the development of antigen-specific therapy for tolerance induction into the clinical setting. Furthermore, it is uncertain whether the delivery of antigen to an already impaired immune system [9–11] is able to correct the autoimmunity.

Dendritic cell therapy provides an alternative way of delivering antigen by using ex vivo-generated cells engineered to control the direction of the immune response toward a preloaded autoantigenic peptides of interest. We and others have demonstrated that peptide-pulsed immature dendritic cell (DC) therapy prevents T1D in NOD mice, the autoimmune diabetes mouse model, when applied during the early stages of autoimmunity [12, 13]. Interestingly, protection from unpulsed DC therapy has also been reported [14–18], challenging the need for antigen. Whether these protective DCs pick up autoantigen *in vivo* or exert antigen-independent influences to the immune repertoire is unknown as most studies using DC therapy have only assessed antigen-specific changes. The global effect that DC therapy may have on nontarget immune cell populations has not been fully elucidated. Moreover, the requirement for early intervention would preclude most patients from its benefits as over 80% of T1D subjects lack familial evidence and do not seek treatment until symptomatic when autoimmunity is well-developed, thereby missing the critical window for early intervention. Thus, an approach that can be initiated within a wider window of time will be more reliable for T1D intervention, and a better understanding of both antigen-dependent and antigen-independent effects of DC therapy will assist in predicting the clinical outcome of DC therapy.

In T1D, T cell reactivity is initially limited to a few autoantigen determinants. However, as disease progresses, autoreactivity gradually expands intra- and intermolecularly to additional determinants and antigens, chronically recruiting naïve cells into the autoreactive pool and possibly leaving an altered immune repertoire with time, providing an explanation for why we observe the fall in efficacy of Ag-based therapies as the rise in autoimmunity expands [19–24]. This epitope spreading gives rise to an array of determinants that have distinct immunogenic properties and possibly unique roles in autoimmune pathogenicity. Regions within the whole antigen that T cells intrinsically recognize and respond to due to preferential antigen processing and presentation by antigen-presenting cells are known as dominant determinants (DD), while subdominant (SD) and ignored (ID) determinants are regions that are minimally unprocessed and unseen and fail to impact the naïve T cell repertoire. As autoreactivity expands to multiple determinants with time, it is expected that fewer T cells remain naïve to DD as they become recruited into a preprogrammed autoreactive response when challenged with a DD. In contrast, even in a late-stage disease, the naïve T cell pool should continue to remain nonreactive to SD or ID as they have had a minimal effect on the naïve T cell pool [25, 26]. Thus, DD-reactive T cells are progressively drained from the naïve pool, while uncommitted naïve T cells remain available to be potentially primed into regulatory function by SD and ID even at later stages of autoimmunity. Olcott et al. first examined this theory by treating NOD mice with a panel of control and T1D-specific autoantigen peptides during late-stage autoimmunity. They showed that only ID, but not target determinants (DD), could protect these mice from diabetes and that the ability of ID to prime Th2 responses did not attenuate with time [26].

In the present study, we hypothesized that through DC-guided presentation of SD or ID, we could better control the direction of the immune response to autoantigen challenge and quench established DD autoreactivity through regulatory T cell-biased bystander suppression. We investigated how various determinant peptides presented through immature DC therapy affected disease outcome when DC therapy was administered to NOD mice with active autoimmunity. In addition, we demonstrated antigen-independent effects of DC therapy and characterized changes in the overall immune response. The findings in this study will contribute to our current understanding on the role of antigen in DC-based therapies and guide the development of DC-based T1D immunotherapy.

## 2. Materials and Methods

### 2.1. Animals

Female NOD/ShiLtj (NOD), C57BL/6J (B6), and Balb/c mice were purchased from The Jackson Laboratory or Animal Care Services at the University of Florida. Bone marrow donor mice were 5–8 weeks of age. Up to five mice were housed together in micro isolator cages in a specific-pathogen-free (SPF) facility with access to food and water ad libitum. Mice were allowed to acclimate to the housing facility for one week prior to the initiation of any studies. Development of diabetes was monitored through twice weekly urine glucose testing using urine glucose test strips (Clinistix, Bayer). Upon detection of glucosuria, a small amount of blood was collected by pricking the tail vein and testing blood glucose using the Accuchek OneTouch glucose meter. A mouse with 2 consecutive daily readings of blood glucose greater than 250 mg/dl was considered to be diabetic. Mice were euthanized by CO_2_ asphyxiation. All mouse experiments were performed in accordance with the University of Florida Institutional Animal Care and Use Committee.

### 2.2. Bone Marrow-Derived Dendritic Cells: Culture and Isolation

The femur and tibia were removed from mice and cleaned of muscle and connective tissue. The ends of the bones were cut, and bone marrow (BM) cells were flushed out with media using a 25–5/8 gauge needle attached to a syringe. Red blood cells were removed from bone marrow cells using ammonium chloride potassium (ACK) lysis buffer for 2 minutes at room temperature, then washed free of lysis buffer using PBS. BM-derived DCs were cultured in RPMI 1640 (Cellgro) supplemented with 10% fetal calf serum (FCS) (Invitrogen Life Sciences), 1x penicillin/streptomycin/neomycin (Gibco), and 10 mM HEPES buffer (Gibco) at a concentration of 10^6^ cells/mL in flat-bottom 6-well culture plates (Corning). 500 U/mL GM-CSF (R&D Systems) and 1000 U/mL IL-4 (BD Pharmingen) were added to BM cultures to promote differentiation into DC. On day 2 or 3, half of the media was replaced with fresh media and cytokines. On day 5 or 6, cells were removed from the bottoms of wells with gentle pipetting and a cell scraper. DCs were purified using CD11c + positive selection magnetic beads (Miltenyi Biotec) and confirmed by flow cytometry to exceed 90% purity. Baseline expression of MHCII, CD80, and CD86 compared to DC stimulated for 24 h with TNF-*α* (semimature) or LPS (mature) was assessed by flow cytometry to characterize maturation state (Supplemental Figure 1).

### 2.3. Dendritic Cell Therapy

Dendritic cells for injection were derived from the bone marrow precursor cells of nondiabetic 4-–8-week-old female NOD mice. 100,000 DCs were suspended in 100 *μ*l of sterile PBS for subcutaneous injection into the area of the hind footpads at 50 *μ*l per footpad. Three weekly injections of PBS or peptide-pulsed or peptide-unpulsed DC (10^5^ cells/mouse) were given to female NOD mice beginning at 9 weeks of age. Mice in short-term treatment studies were treated with one DC injection per week for three weeks, while mice in long-term treatment studies received the short-term treatment followed by boosters every other week. Boosters contained either 200 ng of corresponding peptide in PBS vehicle, or peptide-pulsed DC as received previously. Mice were monitored for normal locomotor activity following footpad injections to ensure no disruption of accessibility to food and drink.

### 2.4. Flow Cytometry

Cells were prepared into single-cell suspensions in FACS buffer (1x PBS/1% FCS) and blocked in Fc Block CD16/32 (2.4G2). Antibody used to identify dendritic cells was CD11c (HL3). Antibodies used to characterize DC maturation were I-A^b^ [25-9-17], I-A^d^ (39-10-8, cross reacts with NOD I-A^g7^), CD80 (16-10A1), and CD86 (GL1). Antibodies used to characterize T cells were CD3 (145-2C11), CD4 (RM4-5), and CD8a (53-6.7). Antibodies used to characterize B cells were B220 (RA3-6B2) and CD19 (1D3). We also used CD25 (PC61) and Foxp3 (FJK-16s) to assess regulatory T cell population, CD11b (M1/70) to assess macrophages, CD44 (IM7) and CD62 (MEL-14) to assess memory T cells, CD138 (281-2) for plasma cells, and CD80 (1610-A1) and CD35 (8C12) for memory B cells. Cells that were further examined for intracellular markers were fixed using Cytofix/CytoPerm reagent (eBioscience) for 15 minutes at room temperature, then washed in Perm/Wash (eBioscience). All subsequent steps were performed in Perm/Wash to maintain membrane permeability. Cells were analyzed by flow cytometer (FACS Calibur, BD Pharmingen). Live cells were gated from dead cells on the basis of forward/side scatter or with 7AAD (amino-antimycin D) labeling. Isotype controls include mouse IgG3*κ*, rat IgG2a, hamster IgG1*κ*, and hamster IgG1*λ*. All antibodies were purchased from BD Pharmingen or eBiosciences. FACS Calibur equipment (BD Biosciences) was used to collect flow cytometry data, and results were analyzed using FCS Express (De Novo).

### 2.5. Peptides

Peptides were purchased from Peptides International (Louisville, KY) and Bio-Synthesis Inc. (Lewisville, TX) and determined to be >90% purity by HPLC analysis. All peptides are tested to be endotoxin-free. Lyophilized peptides were dissolved in RPMI media at 1 mg/mL, then sterile filtered using a syringe apparatus (Gibco). Once resuspended in media, peptides were stored at 4°C as a working solution for up to 2 months. Lyophilized peptides were stored at −20°C indefinitely. Dominant determinants (DD) used were insulin *β*9-23 (SHLVEALYLVCGERG), and subdominant determinant (SD) used was GAD65_78-97_ (KPCNCPKGDVNYAFLHATDL). Ignored determinant (ID) used was GAD65_260-279_ (PEVKEKGMAALPRLIAFTSE).

### 2.6. Dendritic Cell Peptide Pulsing

DCs were pulsed with 3 *μ*M of peptide in cRPMI for 1-2 h in a humidified incubator 37°C with 5% CO_2_. Cells were washed 3 times and resuspended in PBS at 10^6^ cells/mL for injection.

### 2.7. Proliferation Assay

Suspensions of spleen cells were in serum-free HL-1 media (Biowhittaker Cambrex) with the addition of penicillin/streptomycin/neomycin (Gibco) and L-glutamine (Gibco) in triplicate with a selected peptide (25 *μ*M). Cells were cultured at 1 × 10^6^ cells/well in round-bottom 96-well plates at 37°C. At 72 h of culture, 1 *μ*Cu 3H-thymidine (Amersham Biosciences) in 50 *μ*l of media was added per well and allowed to incorporate for 12–16 h. Cells were harvested and washed using an automated cell harvester (Perkin Elmer), and radioactivity was analyzed using a liquid scintillation counter. cpm outliers identified by Grubbs test were removed from analysis.

In assessment of *in vitro* spontaneous proliferation of Tregs following DC therapy, CFSE-labeled spleen cells from female NOD mice from different groups were cultured in serum-free HL-1 media without stimulation and allowed to proliferate for 72–84 h. Cells were subject to surface staining for CD4 and subsequent intracellular staining for Foxp3 and analyzed for proliferating Foxp3+ cells on gated CD4+ T cells.

For assessment of whether IL-2, IL-7, or IL-15 was responsible for the *in vitro* spontaneous T cell proliferation of spleen cells from DC-treated mice, spleen cells were cultured at 1 × 10^6^ cells/well in round-bottom 96-well plates at 37°C in serum-free HL-1 media without stimulation in the presence of isotype IgG antibody, or neutralizing anti-IL-2 antibody, anti-IL-7 antibody, or anti-IL-15 antibody for 84 h. Thereafter, 1 *μ*Cu 3H-thymidine (Amersham Biosciences) in 50 *μ*l of media was added per well and allowed to incorporate for 12–16 h. Cells were harvested and washed using an automated cell harvester (Perkin Elmer), and radioactivity was analyzed using a liquid scintillation counter.

For evaluating homeostatic proliferation in normal and autoimmune mouse models, NOD, B6 mice were treated with 3 weekly subcutaneous injections of DC (10^5^/injection) or PBS beginning at 9 weeks of age, and Balb/c mice at the same age were treated with 3 weekly intravenous injections of DC or PBS. Spleen cells were prepared 2 weeks following final injection to assess 3H-thymidine proliferation in the HL-1 media in the absence of *in vitro* stimulation.

### 2.8. Suppressor Assay

Spleen cells were prepared and suspended in MACS buffer. CD4+ cells were enriched through depletion of unwanted cells using the CD4+CD25+ Regulatory Cell Isolation Kit (Miltenyi Biotec). Next, CD25+ cells were positively selected from the preenriched fraction following the instruction from the manufacturer (Miltenyi). Suppressor CD4+CD25+ cells were cultured with CD4+CD25+ depleted cells (10^5^) at 0 : 1, 1 : 2, and 1 : 4 ratios in a round-bottom 96-well plate. Cells were cultured in serum-free HL-1 media with anti-CD3e (0.05 *μ*g/200 *μ*l well). At 72 h of culture, 1 *μ*Cu 3H-thymidine (Amersham Biosciences) in 50 *μ*l of media was added per well and allowed to incorporate for 12–16 h. Cells were harvested and washed using an automated cell harvester (Perkin Elmer), and radioactivity was analyzed using a liquid scintillation counter. The suppression rate = (proliferation (cpm) without CD4+CD25+ T cells − proliferation (cpm) with CD4+CD25+ T cells)/proliferation (cpm) without CD4+CD25+ T cells.

### 2.9. ELISA for Global Suppression Analysis

Eight-week-old female NOD mice received PBS, unpulsed, or peptide-pulsed DC injections as described previously, once weekly for three consecutive weeks. One week following the last injection, mice were immunized in the footpad with 100 *μ*g/mouse of Keyhole limpet hemocyanin (KLH) (Calbiochem) in Alum (Pierce) weekly for two weeks. Ten to fourteen days following the final KLH immunization, serum samples were collected from mice for the detection of antibodies to KLH by ELISA (Life Diagnostics).

### 2.10. BrdU Incorporation to Assess *In Vivo* Immune Cell Homeostatic Proliferation

Mice received daily intraperitoneal injections of BrdU (bromodeoxyuridine) in sterile PBS (2 mg/100 *μ*l/mouse) for 4 days, then sacrificed 1-2 days following final injection to tissue for analysis of BrdU incorporation. Spleens, livers, and pancreata were fixed in 10% formalin at room temperature for 24–48 hours. Tissues were embedded in paraffin and sectioned at 4 *μ*m for staining using anti-BrdU-HRP Ab and DAB detection and counterstained with hematoxylin. Two sections per sample were collected 100 micron apart for analysis using Aperio's Spectrum ScanScope imaging software. The frequency of BrdU-positive cells was determined using ScanScope's image analysis algorithm that detects positively stained cells on the basis of programmed color and saturation sensitizers within a measured tissue area. Percent BrdU positive is calculated as area positive/area total.

### 2.11. Statistical Analysis

Data were analyzed using the Kaplan–Meier survival curve with Gehan-Breslow-Wilcoxon test to determine if treatment provided protection. Student's *t*-test was also used to identify statistical differences. The Grubbs' test identifies outliers in triplicate wells of proliferation assays. A criterion of *p* < 0.05 was used to define significance.

## 3. Results

### 3.1. Bypassing of Natural Antigen Processing Using DC Pulsed with Underpresented Autoantigen Peptides Leads to T1D Protection in NOD Mice with Active Autoimmunity

**Figure 1:**
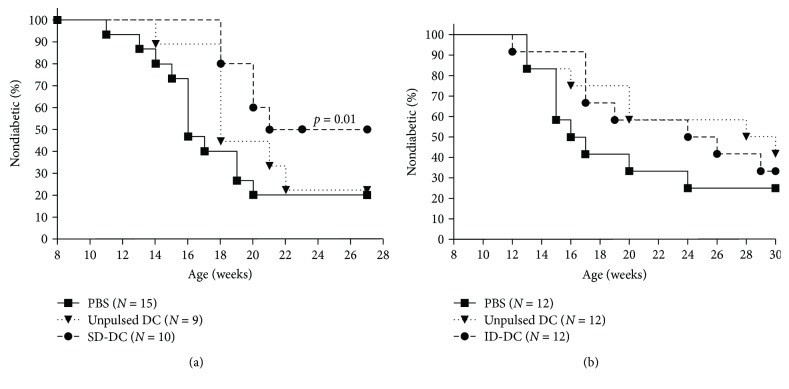
Injections of immature DCs pulsed with subdominant and ignored dominant *β* cell antigenic peptides significantly delay T1D in NOD mice. (a) Nine-week-old NOD mice received subcutaneous injection of PBS, unpulsed DCs, or subdominant determinant-pulsed DCs, once a week for 3 weeks. Then, the mice were monitored for diabetes onset till 27 weeks of age. (b) Nine-week-old NOD mice received subcutaneous injection of PBS, unpulsed DCs, or ignored determinant peptide-pulsed DCs, once a week for 3 weeks. Then, the mice were monitored for diabetes onset till 30 weeks of age. Kaplan–Meier survival curves were depicted, and statistical analysis was performed using Log-Rank test; *p* < 0.05 is considered statistically significant.

Antigen-based studies in mice have demonstrated that DD are ineffective for tolerance induction when applied as peptide therapy in NOD mice with progressive insulitis, and emerging data suggest that use of nontargeted determinants may allow better priming of naïve T cells into regulatory function if treatment is initiated after the autoimmune process is well-established [26]. While SD and ID determinants may be able to better prime regulatory responses from naïve T cells, their reduced or lack of constitutive presentation may require lifelong treatment to maintain the regulatory T cell pool. Thus, we first aimed to assess whether short-term DC therapy pulsed with subdominant determinants better protected NOD from T1D compared to unpulsed DC. We treated 9-week-old female NOD mice with three treatments of bone marrow-derived immature DC unpulsed, or pulsed with synthetic peptides of SD. As shown in Figure 1(a), we found that only recipients of SD-DC, but not PBS or unpulsed DC, were protected from T1D (*p* = 0.01). Of note, SD-DC were able to significantly delay T1D in 100% of SD-DC recipients through the 17th week of age while 40% of PBS controls became diabetic. This suggests that complete protection was conferred for over 8 weeks, and the protection was not durable for the life of the animal. However, complete protection would be ideal in the clinical setting. As ID do not naturally elicit T cell responses, we hypothesized that a larger pool of naïve T cells responding to ID would remain available for priming into tolerance compared to SD. This advantage in available naïve T cell pool size may translate into better protection. Therefore, we performed another study using ID-pulsed DC in 9-week-old NOD mice with active autoimmunity. We administered three weekly injections of PBS, unpulsed, or ID-pulsed DC to mice and observed them for the development of T1D. Surprisingly, we found that ID-DC treatment was not able to significantly protect mice from T1D though we did observe an initial delay in T1D development (Figure 1(b)).

### 3.2. Repetitive Administration of DC Pulsed with ID or SD Prolongs T1D Protection

**Figure 2:**
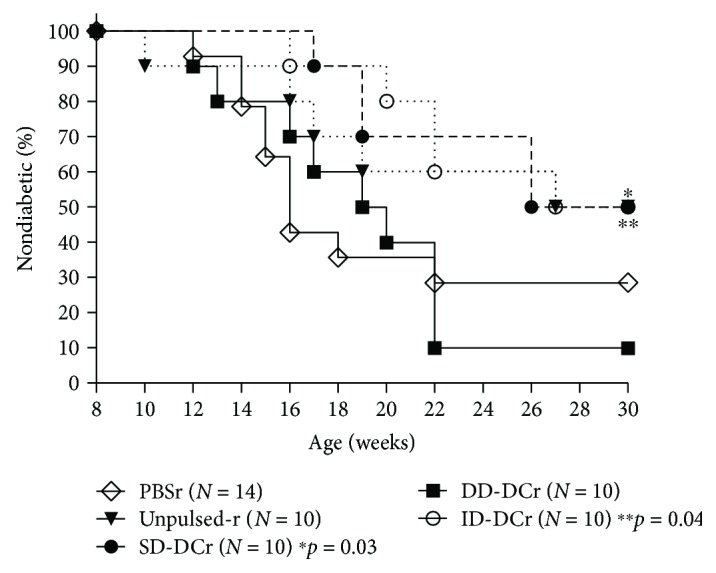
DC therapy-induced T1D protection can be maintained by ignored or subdominant determinant antigenic peptide boosters. Nine-week-old NOD mice received three weekly injections of PBS, unpulsed DCs, or DCs pulsed with DD, SD, or ID peptides. Thereafter, the mice received the corresponding treatment every other week until the study ended. Diabetes onset was monitored once a week. *P* values represent difference compared between PBS and treatment groups.

To assess whether the lack of constitutive presentation of the ID accounted for the loss, we refined this study to include repetitive injections that allowed for consistent presentation of the normally unpresented determinants. Since cell procurement in the clinical setting is both costly and labor-intensive, we wanted to first elucidate whether peptide-only boosters following short-term peptide-pulsed DC could maintain the protection. Because the fate of peptide therapy in the absence of a DC carrier is unknown, in a separate group of mice, we also followed the initial short-term priming treatment with peptide-pulsed DC boosters as proof of principle to account for any peptide competition that may occur *in vivo*. Boosters were given every other week until the end of the study. We found that peptide-only boosters could not continue protection (data not shown). However, as shown in Figure 2, repetitive SD (*p* = 0.01) or ID-pulsed DC treatment was protective (*p* = 0.03) in contrast to PBS control group. No protection was observed in mice receiving repetitive PBS or DD-pulsed DC treatment.

### 3.3. T1D-Specific Peptide-Pulsed DC Therapy Does Not Alter Immune Response to Non-T1D Antigen Challenge in Terms of Development of Antigen-Specific Antibodies

**Figure 3:**
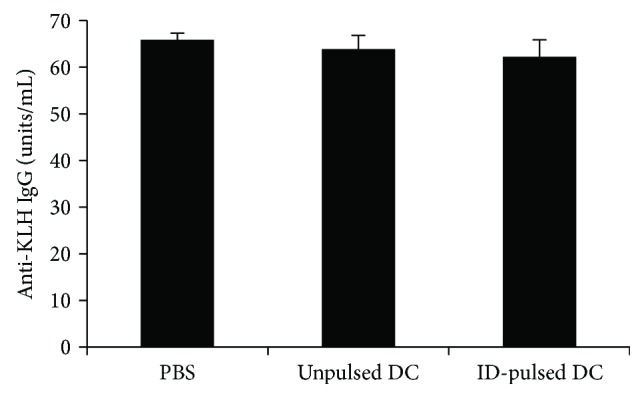
Antibody response following KLH immunization in control and DC-treated mice. NOD mice were treated with 3 weekly injections of either PBS or unpulsed DCs or ID-pulsed DCs (*N* = 3/group). Two weeks following DC therapy, mice were immunized with 2 weekly injections of KLH. Serum antibody levels were assessed 14 days following final KLH immunization. The levels of anti-KLH antibodies of each group are shown as mean ± SD.

Because we observed an initial delay in development of T1D in all mice receiving DC therapy, we were uncertain whether the apparent DC-induced protection against T1D was actually due to an overall dampening of the immune response. We sought to evaluate whether DC therapy conferred specific protection against T1D, or whether the observed protection was an artifact of global immunosuppression that renders mice tolerant to all immune challenges. We tested this by evaluating the ability of DC-treated mice to respond to a non-T1D-specific antigen challenge. We administered either PBS, unpulsed, or ID-pulsed DC therapy as described previously, then immunized the mice with keyhole limpet hemocyanin (KLH), a protein commonly used to examine and elicit immune responses. Two weeks following KLH immunization, we collected sera from the treated mice to detect if an antibody response was mounted against KLH. As shown in Figure 3, there was no difference in the ability of DC-treated mice to generate an antibody response to KLH challenge as compared to PBS-treated mice (*p* > 0.05), suggesting that normal immune processes were intact and the protection previously observed can be attributed to T1D-specific protection.

### 3.4. Homeostatic Lymphocyte Proliferation Is Observed following DC Therapy: Immediate and Sustained Effects

**Figure 4:**
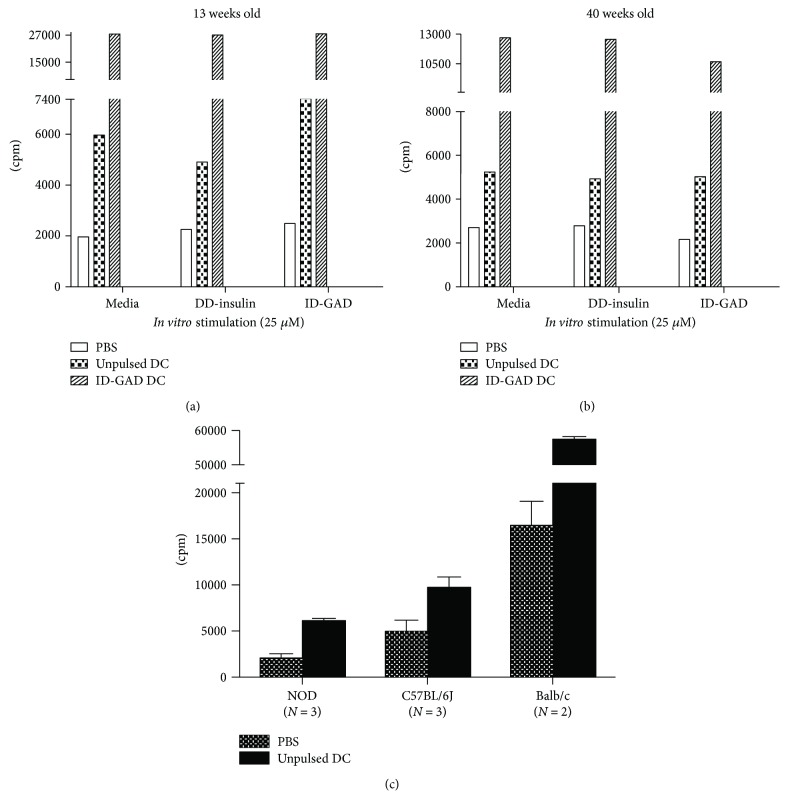
Spleen cell homeostatic proliferation following DC therapy. (a) Nine-week-old NOD mice received subcutaneous injection of PBS, unpulsed DCs, or ID peptide-pulsed DCs, once a week for 3 weeks. Spleen cells from NOD mice of each group at 13 weeks of age were cultured in serum-free HL-1 media alone or with DD-insulin, or ID-GAD for 86 h, and 3H-thymidine was added for incorporation during the final 16 h of culture. Proliferation was assessed by liquid scintillation quantification of counts per minute (cpm). Data shown are the mean cpm (counts per minute) of triplicate values from one of ten experiments. (b) Nine-week-old NOD mice received subcutaneous injection of PBS, unpulsed DCs, or ID peptide-pulsed DCs, once a week for 3 weeks. Spleen cells from NOD mice of each group at 40 weeks of age were cultured in serum-free HL-1 media alone or with DD-insulin, or ID-GAD for 86 h, and 3H-thymidine was added for incorporation during the final 16 h of culture. Proliferation was assessed by liquid scintillation quantification of counts per minute (cpm). Data shown are the mean cpm (counts per minute) of triplicate values from one of ten experiments. (c) Homeostatic proliferation was observed in healthy and autoimmune mouse models. NOD, B6 mice were treated with 3 weekly subcutaneous injections of DC (10^5^/injection) or PBS beginning at 9 weeks of age, and Balb/c mice were treated with intravenous injection of DC or PBS at the same age. Spleen cells were collected 2 weeks following final injection to assess 3H-thymidine proliferation in the HL-1 media in the absence of *in vitro* stimulation. Data shown are the mean cpm (counts per minute) ±SD.

In our studies, we observed that antigen pulsing with SD or ID determinants improved disease outcome. However, mice receiving unpulsed DC also seemed to exhibit a delay in T1D development though they did not achieve significant protection. Since protection from unpulsed DC therapy has been reported in early intervention studies, we wanted to assess how DC therapy affected the immune response as a whole including antigen-independent responses. The spleen is a major site of immune cell interactions and antigen processing, with active processes that contribute to the overall immune status [27, 28]. Thus, we sought to examine cellular responses in this immune cell-rich environment. To evaluate the spleen cell response following DC therapy, we cultured spleen cells with and without autoantigen peptide stimulation for 86 hours, then observed for proliferation using 3H-thymidine incorporation. We found that even in the absence of *in vitro* peptide stimulation, spleen cells isolated from all DC-treated mice had 3–14-fold increase in proliferation compared to PBS-treated mice (Figure 4). This effect of spontaneous proliferation was enhanced in mice receiving antigen-pulsed DC but did not increase with recall peptide challenge suggesting that the response was not eliciting a pathogenic reactivity to the immunizing peptide. The proliferation was seen as soon as just 2 weeks following the last DC treatment at 14-week age (Figure 4(a)) and continued into 40 weeks of age, 29 weeks after the cessation of treatment (Figure 4(b)).

### 3.5. Homeostatic Proliferation Occurs in Healthy and Autoimmune Mouse Strains following DC Therapy

Homeostatic proliferation has been linked to immunodeficiency which promotes a compensatory expansion of “immunological space” [29]. Because NOD mice have been shown to have abnormalities in the immune function of many cell types including differences in DC phenotype and function [30–34], we evaluated whether this homeostatic proliferation was a true effect of DC therapy or only an effect associated with immunotherapy in an animal afflicted with aberrant immune cell subsets. We administered DC therapy to the autoimmune NOD mouse model as well as the healthy control mouse models C57BL/6J and Balb/c and evaluated spleen cell proliferation. As depicted in Figure 4(c), spleen cell homeostatic proliferation following DC treatment occurred in both NOD and nonautoimmune-prone mouse models, suggesting that DC therapy uniquely resulted in a reprogramming of immune cell homeostasis. Additionally, this pattern was independent of route of administration, as Balb/c mice were treated with intravenous DC injections while NOD and B6 mice were given subcutaneous injections.

### 3.6. Homeostatic Proliferation Is Driven by Interleukin-2

**Figure 5:**
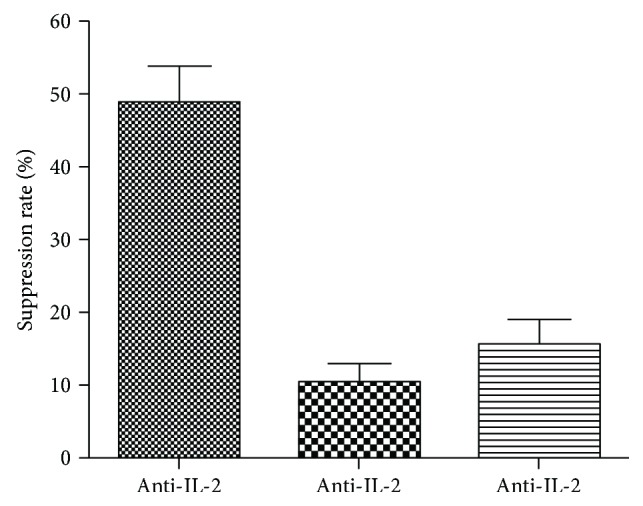
IL-2 is a contributing cytokine for the proliferating cell populations. Spleen cells prepared from different groups shown in the figure were cultured in serum-free HL-1 media for 86 hours in the presence of isotype control Ab, or neutralizing Ab against IL-2, IL-7, or IL-15. 3H-thymidine was added to culture for the last 16 hours. Data shown is representative of 10+ experiments collected through a range of posttreatment time points (13–41 weeks of age).

Our experiments comparing NOD mice to healthy control C57BL/6J and Balb/c mice revealed that spontaneous proliferation following DC treatment is not attributed to lymphopenia possibly happening in NOD mice. Flow cytometric phenotyping of the proliferating cells did not provide evidence for CD4+CD44^hi^CD62^lo^ memory T cell nor CD80+CD35+ memory B cell expansion (data not shown). Another mechanism driving the expansion may be soluble cytokines that contribute to proliferation or maintenance of homeostasis. Studies have shown that IL-2 and IL-15 can activate NK, T, and B cells, induce their proliferation and survival, and stimulate cytokine production [35, 36]. IL-7, a related cytokine sharing the common gamma chain, has been shown to have a role in T cell development, homeostatic proliferation, and survival [35, 37, 38]. Thus, we performed proliferation assays in the presence of cytokine neutralizing antibodies to assess whether proliferation could be abated. We found that neutralization of IL-7 or IL-15 had a minor effect on cell proliferation, while culture with IL-2 neutralizing antibody significantly reduced the expansion of spleen cells of DC-treated mice by 45%, twice the effect observed from cells of PBS-treated mice (Figure 5).

### 3.7. DC Therapy Results in Sustained Expansion of Regulatory T Cells with Enhanced Immunosuppressive Function

**Figure 6:**
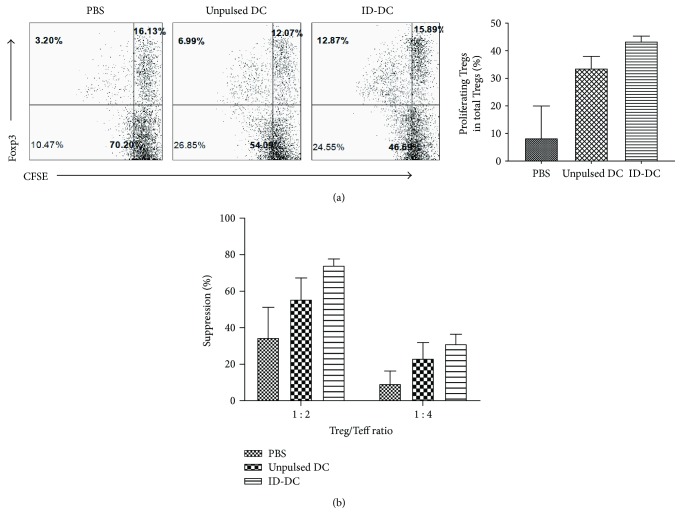
Assessment of Treg spontaneous proliferation and function induced by DC therapy. (a) Assessment of *in vitro* spontaneous proliferation of Tregs following DC therapy. CFSE-labeled spleen cells from female NOD mice from different groups were cultured in serum-free media without stimulation and allowed to proliferate for 72–84 h. Cells stained with CD4 and Foxp and analyzed by flow cytometry. The proliferating Foxp3+ cells were analyzed by gating on total CD4+ cells. Data shown is representative of 3 experiments from mice aging from 13–41 weeks old. (b) For suppressor T cell assay. Female 9-week-old NOD mice were treated with 3 weekly injections of DC, then Treg function was assessed at 13 weeks of age. CD4+CD25+ Tregs were purified and cocultured with CD4+CD25+ T cell-depleted spleen cells at ratios of 0 : 1, 1 : 2, and 1 : 4 and stimulated with anti-CD3 antibodies (0.05 *μ*g/ml). Proliferation was assessed by 3H-thymidine incorporation. The suppression rate = (proliferation (cpm) without CD4+CD25+ T cells − proliferation (cpm) with CD4+CD25+ T cells)/proliferation (cpm) without CD4+CD25+ T cells.

Our work has shown that DC therapy protects mice from T1D and induces noninflammatory homeostatic proliferation of CD4+ T cells. Evidence from the literature suggests that a possible mechanism for protection from DC therapy is the induction of regulatory T cells. Thus, we sought to examine whether Tregs are being induced and whether they are part of the proliferating cell population. Following unpulsed and antigen-pulsed DC therapy, we looked for changes in regulatory T cell frequency and function by evaluating the proportion of CD4+Foxp3+ cells in DC-treated and control mice and examining their ability to suppress proliferation of effector cells. As shown in Figure 6(a), we found that there was an over 2-fold increase in the frequency of CD4+Foxp3+ T cells in mice receiving unpulsed DC and an over 4-fold increase in frequency of CD4+Foxp3+ T cells in mice receiving ID DC, demonstrating that DC therapy resulted in sustained expansion of regulatory T cells and that the effect was particularly enhanced in mice receiving ID-pulsed DC. This homeostatic expansion of Tregs was independent of *in vitro* peptide stimulation, as the pattern was observed in both stimulated (data not shown) and unstimulated cell cultures.

We also examined whether there were functional differences in regulatory T cells following DC therapy. We performed a suppressor cell function assay by coculturing CD4+CD25+-depleted spleen cells with CD4+CD25+-purified cells at ratios of 0 : 1, 1 : 2, and 1 : 4 in the presence of anti-CD3. As seen in Figure 6(b), regulatory T cells from both unpulsed and peptide-pulsed DC-treated mice demonstrated greater suppressive function in a dose-dependent manner, with the effect enhanced in the peptide-pulsed DC group. The enhanced suppression was found to be nearly 2-3-fold greater in DC-treated mice at a 1 : 2 ratio. This effect was magnified when the ratio was decreased to 1 : 4, where up to a 10-fold enhancement in suppression was observed. These results demonstrate that on a cell-to-cell level, regulatory T cells isolated from DC-treated mice are more potent in suppressor function than those isolated from PBS-treated mice.

## 4. Discussion

T1D is a dynamic autoimmune disorder characterized by T cell-mediated destruction of pancreatic islets driven by an expanding T cell autoreactivity toward *β* cell autoantigens. Dendritic cells, which present antigen and direct T cell responses, are an ideal platform for use in T1D treatment as DC therapy could potentially correct the specific underlying autoimmune aberrancy in T1D. DC therapy can uniquely control (1) the direction of the immune response through the selection of either immunogenic or tolerogenic classes of DC, as well as (2) dictate the target antigen that the response is directed toward through the presentation of a chosen antigen, reinforcing DC therapy to be an effective and powerful strategy for immune modulation. Reports of DC therapy for tolerance induction have been successfully demonstrated when applied before or in the early stages of autoreactivity in animal models of various autoimmune diseases, as well as in studies of transplant/graft acceptance [39–45]. However, if treatment is initiated after the autoimmune process is active, efficacy in DC-mediated protection declines. While NOD mice have a predictable timeline for T1D onset allowing for intervention to be planned accordingly, the dynamics of autoreactivity processes in human has been difficult to define due to multiple variations in subtypes that compound assessment. Additionally, the majority of subjects susceptible to T1D lack familial history that would otherwise prompt early autoantibody screening; thus, the opportunity for early intervention in humans is low, emphasizing the need for therapy that can treat both established and new onset disease.

We sought to understand how to better develop DC therapy for translation into the clinical setting. To create DC for therapy with more durable protection, we considered another aspect of DC therapy: selection of antigen for loading prior to infusion. We and others have demonstrated that the administration of *β* cell autoantigens in a tolerogenic modality is highly effective in preventing T1D in the NOD mouse [1, 2, 22, 46–49]. However, uncertainties in extrapolating appropriate antigen doses and correlating treatment timeline have hindered its translation into the clinical setting, particularly since studies have shown that the immune response can pivot toward immunity or tolerance depending on antigen dose. Fortunately, antigen presentation in the context of a tolerogenic DC may circumvent the issue of ambiguous immune deviation associated with antigen treatment alone. Based on work from Kaufman's group, we believed that dominant determinants (DD) identified to be the initiators of the autoimmune response chronically recruit naïve T cells into the pathogenic pool; thus, the readministration of these determinants only reactivated cells that were programmed to respond pathogenically [26]. However, subdominant determinants (SD) or ignored determinants (ID), which have a minimal impact on naïve T cell activation, should have large pools of naïve T cells available for priming into tolerance when we bypass natural antigen processing to experimentally present these peptides. We compared the efficacy of DD, SD, and ID peptide classes in DC therapy to protect 9-week-old NOD mice and found that only SD- and ID-pulsed DC were able to protect mice when the treatment was applied in NOD with active ongoing autoimmunity (Figure 2). Specifically, just three weekly injections of 1 × 10^5^ SD-DC protected NOD from T1D with a significant delay in the onset of T1D, though complete protection was not achieved. We examined whether ID-DC, which should have a comparatively larger pool of naïve T cells to prime into tolerance, would be more effective in conferring protection. However, we found that three injections of ID-DC were not sufficient to achieve prolonged protection, as a sudden increase in diabetes onset within 6 weeks of the last treatment dampened the treatment success. We speculated that since ID are not constitutively presented, treatment may need to be continued to maintain the regulatory T cell pool. We treated another cohort of mice as previously described, then followed by a series of priming injections with boosters every other week. We found that mice receiving boosters of SD-DC or ID-DC were significantly protected from T1D. This enhanced protection was not seen in mice treated with repetitive injections of PBS or unpulsed or DD-pulsed DC, suggesting that the protection is attributed to the nature of the antigens. Because cell procurement is a labor-intensive and costly treatment, we were also interested in determining whether peptide-only boosters following the initial priming series could effectively maintain the same protection. Unfortunately, mice receiving peptide-only boosters following the initial DC priming developed T1D with age (data not shown). It is possible that our selected peptide-boosting dose was not optimal to maintain tolerance, or the peptide presented by the host antigen-presenting cells altered T cell functionality. Alternatively, this finding stresses the importance of the role of DC in therapy. Administration of peptide alone to a host with an existing aberrant immune system may be futile. Likewise, it is unknown what happens to peptide without using DC as an antigen carrier because peptide competition *in vivo* could render the injected peptide irrelevant. Overall, our findings indicate that antigen presentation, and particularly the class of determinant, plays an important role in DC-based immune modulation.

Consistent with this finding, we observed that antigen-DC-treated mice had a greater number of pancreatic islets compared to unpulsed-DC-treated mice (data not shown). We failed to observe *β* regeneration in all groups. Thus, it is possible that the islet preservation we observed was achieved through the induction of regulatory T cells that was enhanced with antigen-pulsed DC treatment, but this treatment was not enough to completely quench the inflammation generated from the pathogenic T cells.

To exclude the possibility that the observed protection from T1D was due to global immunosuppression, we examined whether NOD mice could generate a normal immune response to a non-T1D-related antigen challenge following treatment with DC therapy. We immunized PBS, unpulsed DC, and ID-DC-treated mice with KLH and examined their serum antibody responses. All DC-treated mice were able to mount antibody responses to KLH in a manner comparable to PBS controls, suggesting that normal immune responses were intact and the previously observed protection could be attributed to diabetes-specific protection.

Much of the current knowledge on how DC therapy affects immune responses has been delineated from studies with a focus on antigen-specific immune modulation, as we have shown that antigen-based DC therapy-mediated protection is limited to the suppression of autoreactive processes specific to T1D. However, whether DC therapy results in antigen-nonspecific immune changes has not been well investigated. Recent evidence suggests that *β* cell antigens in T1D immunotherapy might not be necessary for therapy-induced protection [50]. The spleen is a major site of immune cell interactions and antigen processing, with active processes that contribute to the overall immune status [27, 28]. Thus, we sought to examine the spleen cell response in the absence and presence of T1D peptide stimulation. To our surprise, we found robust *in vitro* homeostatic proliferation of spleen cells isolated from DC-treated mice (unpulsed or antigen-pulsed), but not PBS-treated mice. The resulting changes are not antigen specific as we find this reprogrammed spleen cell responses in the absence of antigen stimulation. Furthermore, this effect was immediate and sustained, as the proliferation could be observed as early as just two weeks following only 2 DC injections, and was durable even 29 weeks after treatment had ended. Further characterization of these cells revealed that the proliferation was predominantly attributed to B and T lymphocytes (data not shown). In addition, by screening T cell proliferation-related cytokines in this DC therapy-induced spontaneous proliferation of lymphocytes, IL-2 was found to be the responsible cytokine while IL-7 and IL-15 played a minor role.

Because NOD mice have a defect in DC phenotype and function, we evaluated whether this homeostatic proliferation was a true effect of DC therapy or an only effect associated with therapy using DC with an aberrant phenotype. We treated the nonautoimmune-prone mouse strains Balb/c and C57BL/6J mice with PBS or DC and observed a similar enhancement in homeostatic proliferation in mice receiving DC therapy, confirming that the effect is a true immune response to DC therapy.

To determine whether homeostatic proliferation occurred *in vivo*, we treated mice with BrdU and collected spleens to detect for BrdU incorporation. We were not able to detect a difference in percentage of proliferating cells between mice treated with PBS compared to DC-treated mice (data not shown). We also sought to determine whether the protection observed resulted from increased beta cell regeneration. We examined pancreata and found no differences in BrdU incorporation.

In T1D, the lack of an adequate regulatory response allows autoreactive T cells to become pathogenic, thereby invading and destroying the pancreatic islet cells. Multiple studies have demonstrated that DC therapy can confer protection against autoimmunity through the induction of regulatory T cells that inhibit the pathogenic T cell inflammation [44, 51–64]. Thus, we evaluated the effect of DC therapy on the regulatory T cells. Consistent with our earlier finding of homeostatic CD4+ T cell expansion, we found that DC therapy resulted in a durable 2-3-fold increase in the frequency of proliferating CD4+Foxp3+ regulatory T cells when cultured *in vitro*, and this effect was further enhanced using ID-DC. DC therapy also enhanced the immunosuppressive function of Tregs, as CD4+CD25+ regulatory cells from DC-treated mice more potently suppress anti-CD3 antibody-stimulated proliferation of CD4+CD25-depleted responder cells compared to Tregs from PBS controls. These results suggest that DC (esp. antigen-pulsed) therapy primes generation of more immunosuppressive Tregs. Again, the addition of antigen to DC therapy leads to even greater enhanced suppressive function. Of interest, these findings were observed in both nondiabetic and delayed diabetic mice emphasizing the correlation to DC treatment. While we observed an increase in both frequency and function of Tregs with DC treatment *in vitro*, we did not observe a correlation in protection from T1D *in vivo*. A potential explanation was proposed by work from Diane Mathis's group, which demonstrates that while defects in NOD Tregs contribute to T1D, it may be an effect of overresponsive effector T cells to self-antigen that truly drive the immunopathology [65]. Thus, the loss of tolerance may be related not to impaired function or decreased frequency of NOD Tregs, but rather a decline in the ability of NOD T cell effectors to respond to fully competent Tregs. However, our studies of Treg function examine PBS-treated Tregs versus DC-treated Tregs against NOD effectors, which in concept should be similarly impaired, so our observation of functional differences between the treatment groups can be attributed to a true variation between PBS- and DC-treated Tregs. Alternatively, it is possible that the improvement was not sufficient to pivot the balance in favor of regulation in the presence of a potent inflammatory effector T cell response that has been shown to grow with age [65]. This may be supported by our observation of increased islet survival in antigen-pulsed-DC-treated mice, but not unpulsed-DC-treated mice that have similar levels of lymphocyte infiltrate (data not shown), as the undefined lymphocyte population may potentially be an influx of both pathogenic and regulatory T cells. The influence of regulatory T cells to preserve existing islets must surpass the destruction by pathogenic T cells to maintain physiologically relevant numbers of functional islets for metabolic control of insulin. Nonetheless, the later stages of advanced autoimmunity immediately prior to T1D onset may simply not be amenable to a one-armed intervention; Tregs alone may not be sufficient to rescue *β* cell death and the requirement for combinatorial strategies to treat both autoimmunity and regenerate *β* cell mass may become necessary. To assess the T1D-protective regulatory T cells, an alternative approach to be considered is to use adoptive T cell transfer to examine whether the DC-treatment-induced regulatory T cells are more potent in protecting NOD mice from T1D or protecting NOD-Rag−/− mice from diabetes induced by cotransferred diabetogenic T cells, compared to regulatory T cells from PBS-treated mice.

Collectively, our study demonstrates that DC therapy results in antigen-dependent and antigen-independent effects on immune modulation [66]. We find that the selection of autoantigen peptide for therapies aimed to prime naïve T cells has a critical impact on the efficacy of protection against dynamic autoimmune diseases. Constitutively, underpresented autoantigen determinants may be more effective in tolerance induction when used in late-stage intervention. This fundamental principle of altering native determinant presentation to accommodate a changing T cell repertoire can be extended to the design of treatment for any dynamic autoantigen-based diseases. We also demonstrate that immature DC therapy augments the immune response in an antigen-independent manner resulting in homeostatic expansion of functionally enhanced Tregs. Overall, these findings demonstrate the durable potency of DC therapy in the modulation of antigen-specific and antigen-nonspecific immune responses and provide an important step toward translation into the clinic as other peptide-based therapies for T1D have been limited to early intervention.

## Supplementary Materials

Supplementary Materials Figure 1: characterization of DC phenotype. DCs were differentiated in the presence of GM-CSF and IL-4 for 5-6 days (immature iDC), and baseline expression of MHCII, CD80, and CD86 was assessed by flow cytometry compared with DC stimulated with TNF*α* (semimature smDC) or LPS (mature mDC) for 24 h.
