# Supplementary figures and images for: Immature Dendritic Cell Therapy Confers Durable Immune Modulation in an Antigen-Dependent and Antigen-Independent Manner in Nonobese Diabetic Mice
Source: J Immunol Res. 2018 Feb 14;2018:5463879. doi: 10.1155/2018/5463879 (PMC5832131; doi:10.1155/2018/5463879)

**Supplemental Figure 1**


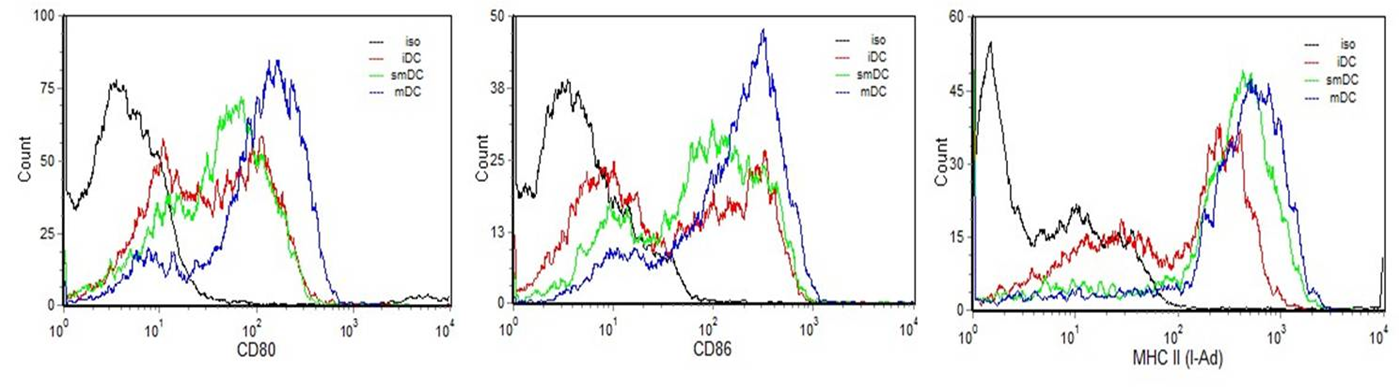

Supplement: Supplementary Materials — Figure 1: characterization of DC phenotype. DCs were differentiated in the presence of GM-CSF and IL-4 for 5-6 days (immature iDC), and baseline expression of MHCII, CD80, and CD86 was assessed by flow cytometry compared with DC stimulated with TNFα (semimature smDC) or LPS (mature mDC) for 24 h. [file 5463879.f1.docx]
